# A study of candidate genes for day blindness in the standard wire haired dachshund

**DOI:** 10.1186/1746-6148-4-23

**Published:** 2008-07-01

**Authors:** Anne Caroline Wiik, Ernst-Otto Ropstad, Ellen Bjerkås, Frode Lingaas

**Affiliations:** 1 Department of Basic Sciences and Aquatic Medicine, Division of Genetics, Norwegian School of Veterinary Science, P.O.Box 8146 Dep, 0033 Oslo, Norway; 2 Department of Companion Animal Clinical Sciences, Norwegian School of Veterinary Science, P.O.Box 8146 Dep, 0033 Oslo, Norway

## Abstract

**Background:**

A genetic study was performed to identify candidate genes associated with day blindness in the standard wire haired dachshund. Based on a literature review of diseases in dogs and human with phenotypes similar to day blindness, ten genes were selected and evaluated as potential candidate genes associated with day blindness in the breed.

**Results:**

Three of the genes, *CNGB3*, *CNGA3 *and *GNAT2*, involved in cone degeneration and seven genes and loci, *ABCA4*, *RDH5*, *CORD8*, *CORD9*, *RPGRIP1*, *GUCY2D *and *CRX*, reported to be involved in cone-rod dystrophies were studied. Polymorphic markers at each of the candidate loci were studied in a family with 36 informative offspring. The study revealed a high frequency of recombinations between the candidate marker alleles and the disease.

**Conclusion:**

Since all of the markers were at the exact position of the candidate loci, and several recombinations were detected for each of the loci, all ten genes were excluded as causal for this canine, early onset cone-rod dystrophy. The described markers may, however, be useful to screen other canine resource families segregating eye diseases for association to the ten genes.

## Background

Inherited retinal degenerations form a diverse spectrum of blinding disorders in humans and other mammals, including a large number of dog breeds [1]. Retinal diseases are inherited as monogenic or complex traits. In the last two decades over 130 genes that cause retinal diseases have been identified [2].

Progressive retinal atrophies (PRA) are the most common retinopathies in dogs and constitute a heterogeneous group of phenotypically similar disorders equivalent to retinitis pigmentosa (RP) in man. PRA, like RP, is primarily a disease of rod photoreceptors, while cone function and structure degenerate secondarily. PRA has been identified and studied in more than 100 breeds and distinct loci primarily responsible for each disorder have been identified in at least 22 different breeds [1,3].

The cone dystrophies comprise a phenotypically heterogeneous group of hereditary retinal degenerations characterized by progressive dysfunction of the photopic (cone-mediated) system. The presenting signs include day blindness, loss of colour vision, reduced central visual acuity and preserved peripheral vision [4]. The cone dystrophies are genetically heterogenous and may be sporadic, autosomal dominant, autosomal recessive or X-linked recessive [2].

Canine cone degeneration is phenotypically similar to human achromatopsia [5]. Identified genes associated with human achromatopsia are the phototransducin genes *GNAT2 *(HSA1) [6], the *CNGA3 *(HSA2) [7] and the *CNGB3 *(HSA8) [8] genes.

Autosomal recessive cone degeneration occurs naturally in Alaskan malamute [9] and German shorthaired pointer (GSP) and is due to different mutations in CNGB3 [5,10]. Sporadic cases is seen in a number of other breeds [11,12].

The cone-rod dystrophies (crd) are characterized by a predominant loss of cone function, with relative preservation of rod function [13,14]. The number of genes or loci currently identified for crd in humans is low, compared to those for RP [2]. Identified genes associated with autosomal recessive inherited crd in man include *ABCA4 *[15,16] and *RDH5 *[17] in addition to the mapped loci *CORD8 *[18,19] and *CORD9 *[20]. *ABCA4 *and *RDH5 *are genes involved in the retinoid cycle, the *CORD8 *and *CORD9 *loci are yet to be identified. The standard wire haired dachshund (SWHD), the miniature long haired dachshund (MLHD) and the pit bull terrier (PBT) are the only dog breeds to date known to be affected by crd [21-23].

Canine *ABCA4 *has been studied as a candidate gene for cone-rod degeneration in the pit bull terrier, although no mutations were found [21]. Mutations in the *RPGRIP1 *gene have been reported to be a cause of Leber congenital amaurosis (LCA) [24,25]. LCA is the earliest and most severe form of all retinal dystrophies responsible for congenital blindness [26]. Mutations causing residual *RPGRIP1 *activity may lead to phenotypes such as RP or crd, which are less severe than LCA [27]. In 2003, Hameed et al. (2003) [28] showed evidence that some *RPGRIP1 *gene mutations are associated with recessive cone-rod dystrophy. Recently Mellersh et al. (2006) [22] found a mutation in canine *RPGRIP1 *associated with autosomal recessive crd in miniature longhaired dachshunds.

A high level of genetic heterogeneity of the disease group is observed and a range of mutations in *RPGRIP1*, *CRX *and *GUCY2D *cause different retinal diseases with different modes of inheritance [14,27-34].

A resource strain of standard wire haired dachshund displaying day blindness was developed to allow characterization of the phenotype and identification of the causal mutation. Electroretinography (ERG) and clinical studies showed that secondary degeneration of the rods appeared during the progression of the disease of the dogs of the colony, indicating a progressive cone-rod dystrophy (crd) [23]. The disease seems to be inherited in an autosomal recessive manner, and diversity in the age of onset and progression of the retinal degeneration within the group of affected dachshunds is observed.

This study was conducted in parallel with clinical studies [35] and genes known to be involved in human cone degeneration, cone dystrophy and cone-rod dystrophy were evaluated as candidate genes for the day blindness in the SWHD (See Table 1).

**Table 1:** Overview of the candidate genes

**Candidate**	**Canine chr**	**Location**	**Annotation**	**Coding for**	Disease*
CNGB3	CFA29	35896147-35752878	NC_006611	cyclic nucleotide gated channel beta 3	cd
CNGA3	CFA10	47377091-47357441	NC_006592	cyclic nucleotide gated channel alpha 3	cd
GNAT2	CFA6	45319301-45327681	NC_006588	guanine nucleotide binding protein (G protein), alpha transducing activity polypeptide 2	cd
ABCA4	CFA6	58112924-58240779	NC_006588	ATP-binding cassette, sub-family A (ABC1), member 4	crd
RDH5	CFA10	3102451-3106432	NC_006592	retinol dehydrogenase 5 (11-cis/9-cis)	crd
CORD8	CFA9	HSA1q12–24		unknown	crd
CORD9	CFA29	HSA8p11		unknown	crd
RPGRIP1	CFA15	21355638-21394395	NC_006597	retinitis pigmentosa GTPase regulator interacting protein 1	crd
GUCY2D	CFA5	35838279-35853509	NC_006587	guanylate cyclase 2D, membrane (retina-specific)	crd
CRX	CFA1	111135799-111146903	NC_006583	cone-rod homeobox	crd

*cd = cone degeneration and crd = cone-rod-dystrophy

## Results

**Figure 1:**
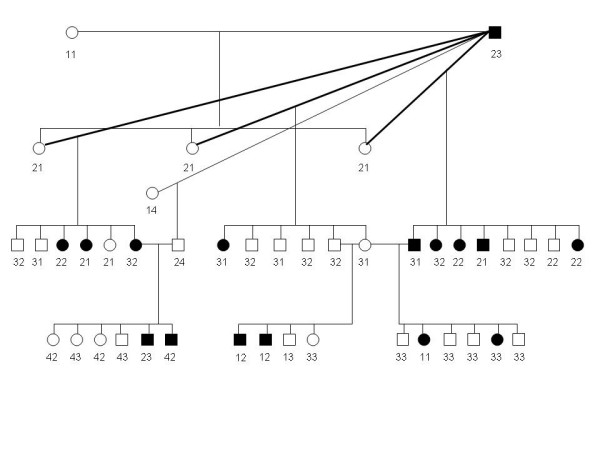
**The informative resource family, consisting of offspring from a single affected male. **The numbers below each individual represent ABCA4 genotypes, and clearly shows the free recombination of marker-alleles compared to disease in the family.

An overview of the studied genes and their location is shown in Table 1. Studies of segregation of each of the 10 candidate loci and clinical disease in informative parents and offspring revealed a number of new candidate gene allele-disease phenotype combinations ("recombinants") in the offspring. The segregation of potential candidate gene alleles was studied in inbred offspring from parents with known disease genotypes. Females where either homozygous affected (= clinical diseased) or carriers (= clinical healthy offspring of affected male). Since the father in these litters usually was homozygous affected, the theoretical phenotype of the offspring would depend on which of the two potential alleles (affected or non-affected) it received from its carrier mother. The most frequently occurring genotype-phenotype combination was counted as the non-recombinants, while any new genotype-phenotype combination, with regard to their parents, was counted as recombinant. Each litter was counted as a separate unit and the recombination frequency were summed over all families. A minimum of 6 recombinants were identified for each of the 10 loci (See Table 2). This excludes all the candidate genes as the cause of mutation causing the disease. The family is illustrated in Figure 1. The genotypes of ABCA4-markers are shown below each individual and show free recombination of ABCA4-variants between affected and unaffected offspring.

**Table 2:** Recombination frequencies between candidate gene loci (microsatellites at locus) and disease

**Locus**	**no. of informative offspring**	**no. of recombinant offspring**	**Recombination frequency**	**LOD score**
CNGB3	34	11	0.32	0.940
CNGA3	20	7	0.35	0.397
GNAT2	31	6	0.19	2.717
ABCA4	34	11	0.32	0.940
RDH5	34	11	0.32	0.940
CORD8	25	10	0.40	0.219
CORD9	18	5	0.28	0.800
CRX	24	8	0.33	0.590
GUCY2D	23	7	0.30	0.786

## Discussion

The large number of retinal genes or loci have been identified and presented at the RetNet™ web page [2], facilitates a candidate gene approach in the study of canine retinal diseases. Human genes with mutations giving similar phenotypes and inheritance patterns to the one found in the affected SWHDs were selected as candidate genes. The preliminary clinical diagnoses for the affected SWHDs suggested that the disease was comparable with human cone degeneration, hence the inclusion of *CNGB3*, *CNGA3 *and *GNAT2*, which are associated primarily with cone degeneration. In the initial phase of the study the family material was not sufficient for segregation studies and we therefore sequenced the coding parts of these three genes. No mutations where identified in any of the three genes (methods and results not shown). Continued electrophysiological and clinical studies of the affected dachshunds were conducted in parallel with the genetic studies and revealed secondary continuing degeneration of the rods, indicating a progressive cone-rod dystrophy (crd) [23]. Seven genes reported to be associated with autosomal recessive cone-rod dystrophies (crd) [2], *ABCA4*, *RDH5*, *CORD8*, *CORD9*, *RPGRIP1*, *CRX *and *GUCY2D *were therefore included in the study.

It is important to be aware of the fact that the disease-allele/haplotype present in all dogs in the family arises from inbreeding on one single affected founder (Figure 1). The informative markers were selected at the exact position of the candidate genes, most of them less than approximately 0.1 Mb distance. A distance of 0.1 Mb compares to a genetic distance of ~0.1 cM, or 1 recombination per 1000 offspring. The minimum number of detected recombinations in our family (1 recombination per 36 informative offspring) would compare to a recombination frequency/genetic distance of ~2.8 cM or a physical distance of ~2.8 Mb. We would not expect any recombinants if a mutation in any of the candidate genes caused this disease with a simple recessive inheritance. Our results show a minimum of 6 recombinations (GNAT2; 31 informative offspring) or more than 19.3 Mb distance (Table 2), excluding all the candidate genes as the site of the mutation causing the disease. However, even though the specific candidate genes can be excluded as the cause of the disease because of the high number of recombinations, the chromosomal regions are not necessarily excluded. The clinical findings defining the diseases as a cone-rod dystrophy rather than cone degeneration also support the exclusion of *CNGB3*, *CNGA3 *and *GNAT2*. The candidate loci *CORD8 *and *CORD9 *are not yet mapped and the genes remain to be identified, but the markers typed for these loci are located within 2.8 Mb of the gene and several recombinations exclude these loci as well.

It might be considered likely that crd in MLDH and SWDH was caused by the same mutation, since these two breeds have in part the same genetic background. There are, however, obvious differences in the clinical phenotypes of the disease in the two breeds. One of the most characteristic clinical findings in the SWDH is pinpoint-sized pupils, observed in 60% of the 8-week old, crd-affected puppies. Older crd-affected dogs displayed dilated pupils and delayed pupillary light reflexes (PLRs) when stimulated with a focal light source [23]. This is in contrast to the clinical signs of PBT and MLHD, which include dilated pupils at 7–8 weeks of age for PBT and normal PLRs at 25 weeks of age for MLHD, respectively [21,34]. The differences in clinical signs between the MLHD and the SWHD, support the finding of free recombination between *RPGRIP1 *and the disease in the SWHD. This shows that this disease in the SWHDs is a unique animal model for cone-rod dystrophy.

The development of animal models of ocular disease represents an invaluable resource for testing and evaluating treatment strategies relevant to both human and canine disease. Attempts at restoring vision in dogs and human by gene therapy have been made, providing optimism regarding potential for recovery of functional vision [36-41]. Uncovering the genetic basis of the cone-rod dystrophy in the SWHD may contribute to finding new gene therapy treatments.

The present work has excluded a number of genes as a cause of crd in the SWHD. Research in human and animals continuously identifies new genes that may be associated with crd. In addition to continued studies of a few specific candidate genes, future research will focus on a whole genome scan by linkage studies with polymorphic markers [42] or by SNP-array typing [43]. The development of the canine genotyping array of ~27000 SNPs show that genome-wide association mapping of mendelian traits in dog breeds can be achieved with only ~20 dogs [44]. The use of SNP array technology, followed by fine mapping based on microsatellites and regional gene studies may be the best method to elucidate which gene is involved in this disease.

## Conclusion

The genes involved in cone degeneration, *CNGB3*, *CNGA3 *and *GNAT2*, and the genes involved in cone-rod dystrophy, *ABCA4*, *RDH5*, *Cord8*, *Cord9*, *RPGRIP1*, *GUCY2D *and *CRX*, were all excluded from being involved in the cone-rod dystrophy described in this family of SWHD. The study provides a number of genetic markers for use in other studies. Further work, based on a whole genome association study will be performed to identify the mutation involved in the disease.

## Methods

### Animals

Day blindness was diagnosed in two wire haired dachshund littermates, one male and one female, in a litter of four. The parents were phenotypically normal. The male was bred to two unrelated crossbred dogs with no history of previous eye disease and the dogs in the F1 generation were unaffected. A purpose-bred colony was established through back-crossing the male with his daughters, producing both affected and unaffected offspring. The retinal changes were always bilaterally symmetrical and the initial onset was observed from 10 months to 3 years of age. A complete retinal atrophy was evident within the age of 5–6 years.

### Clinical diagnosis

To evaluate vision, all dogs were subjected to behavioral testing (maze test), examination of pupillary light reflexes (PLRs), indirect ophthalmoscopy and bilateral full-field electroretinography (ERG). Light microscopy, electron microscopy and immunohistochemistry were carried out in 22 selected cases at ages 5–304 weeks, in order to confirm the diagnoses [23].

All procedures used in this study adhered to the guidelines of the Norwegian Animal Research Authority (Forsøksdyrutvalget) and to the Association for Research in Vision and Ophthalmology's "Statement for the use of Animals in Ophthalmic Vision and Research".

### Samples

Blood samples were collected into EDTA-coated tubes from all the related dogs in the day blind colony and from two non-affected, unrelated dogs. Genomic DNA was isolated from the blood samples using DNeasy Tissue kit (Qiagen, GmbH, Germany).

The collected DNA samples were subjected to candidate gene screening. All the ten loci were typed by polymorphic markers at these loci in five informative dachshund families comprising 43 dogs -7 parents and 36 offspring (Figure 1), to detect recombinants between each of the loci and the disease.

### Study of marker-disease segregation in the resource family

Genotyping of *CNGB3 *was done with two closely linked markers, FH2772 [45] and c29.002 [46], embracing the gene (530 Kb and 350 Kb distance respectively). Markers for genotyping of *CNGA3, GNAT2 ABCA4*, *RDH5*, *CRX*, *GUCY2D *and *RPGRIP1 *were identified by searching a sequence of about 200,000 bp, including the gene(s) (gene +/- ~100 000 bp), for various microsatellite motives. FH2370, a marker closely linked to *ABCA4 *[46] was also genotyped. For the loci *CORD8 *and *CORD9*, 200 000 basepairs surrounding genes at the site of the two loci, respectively *FM05 *and *RP1*, were used to identify polymorphic markers. Tetra- and dinucleotide repeat microsatellites with more than ten and nineteen repeats, respectively, were selected. Primers for amplification of selected microsatellites were designed using primer3 [47] (See Table 3). One primer in each pair was labelled with fluorescein for automated detection in an automated fragment analyzer, ABI3100 Genetic Analyser.

**Table 3:** Primers for amplification of the microsatellites at the exact position of the candidate gene loci

**Microsatellite**	**Forward primer (5'-3')**	**Reverse primer (5'-3')**	**Size of PCR-product (bp)**	**Nucleotide repeat**	**Location (bp)**	**Accession number**
CNGB3-FH2772	CCCAAAGCACATCCTAATTC	GGAGTCTGCTTCTCCCTTTC	168	di	Chr29:35405042-35405217	UniSTS 263646
CNGB3-C29.002	TATTAAATCCCAGTCACCACCC	AGGTCCCAGACCGAGTCC	208	di	Chr29:36423257-36423471	UniSTS 262830
CNGA3-GT19	CCTCCCACTCTCCCCTCTAC	CCAGGGGAGCTTTTACAACA	337	di	Chr10:47307640-47307977	BV729091
CNGA3-GT20	GCAAGCAGTCCCGATTTTTA	TCAGCTTTGGTCATGCACTC	304	di	Chr10:47283009-47283313	BV729076
GNAT2-GAAA16	CCCATGCTTGGTTTAATGCT	GACTGTCCTGCCTTCCATGT	350	tetra	Chr6:45194834-45195184	BV729077
GNAT2-CTT22	CAGCTGGATTCTTCCCATGA	GCCCAAATTGCAAATCCTTA	273	tri	Chr6:45461591-45461864	BV729078
ABCA4-FH2370	CCTGAAAAATAGCTAGATGATGG	GTCTTTACCTGCCTATATAGCTGC	380	tetra	Chr6:58367514-58367919	UniSTS 263571
ABCA4-TTTC16m	GGAATCAGTGGACTCATCCAA	GGGGATTGGACAGTGGTAGA	238	tetra	Chr6:58046419-58046656	BV729079
RDH5-TAGA10	GAAGATGACGATGATGATGAAGA	GCTGAAGGTAGACGCTGGAC	286	tetra	Chr10:3068645-3068931	BV729080
RDH5-GAAA16	CCCTGCTGTGAGGAGTCAGT	AGCCAGATGCAGGACTTGAT	209	tetra	Chr10:3002063-3002276	BV729081
CORD8-FMO5-GAAA12	CCACAAGTTGGGGTTTCAAG	CCCCTCCTCTCTCTCTTTCC	204	tetra	Chr9:60070458-60070662	BV729082
CORD8-FMO5-TTTA10	CCCAGGTGTCCCTATTTTCC	GCTCACTGGGGAGTCTGCT	175	tetra	Chr9:60654305-60654480	BV729083
CORD9-RP1-ATm22	CACTGGATGCACACAGATCC	GGTCCTTGAGAAGGAAGCTG	296	di	Chr29:9128657-9128953	BV729084
CORD9-RP1-TTTC21m	TCCAGTAGGCGTCCTCTGAC	AGTCAATGGAGCCTGCAACT	410	tetra	Chr29:9011174-9011583	BV729085
RPGRIP1-GAAA26	TGTTACCTGTTCCAAAGTTGTTTT	AGTTACAGCCATGGGAATGC	475	tetra	Chr15:21287275-21287750	BV729086
GUCY2D-TTCC15	ACAATGGGCACATCTGTTGA	TTCTCCCTCTGCCTGTGTCT	405	tetra	Chr5:35459311-35459716	BV729089
GUCY2D-GAAA17	CCCCTCTTCTCCACTCTCCT	TCATATTCTTGCCCCAGTCC	444	tetra	Chr5:35514793-35515246	BV729090
CRX-GAAA15m	TGGTCTCACATTCCCACTGA	AGAAGTGGCAGAGCACAGGT	438	tetra	Chr1:111096908-111097346	BV729087
CRX-GT21	ACCAGAACCAAAGGCAGATG	TCAGGGTTGGAGTTTTGAGC	427	di	Chr1:111076912-111077339	BV729088

PCR amplification reactions were performed using 83 μM of each primer in a 15 μl reaction containing 1.5 μl DNA prepared as described above, 1× PCR buffer containing 1.5 mM MgCl_2_, 83 μM each of dATP, dCTP, dGTP and dTTP, and 0,33 units Taq DNA polymerase (Qiagen). After an initial denaturation at 95°C for 2 min and 30 sec, samples were amplified for 28 cycles at 95°C for 30 sec, 58°C for 40 sec and 72°C for 50 sec, followed by a final extension of 72°C for 5 min and 30 sec. The sizes of the alleles were estimated with an automated sequencer (ABI PRISM^® ^3100 Genetic Analyzer, Applied Biosystems, Foster City, CA, U.S.A.) with software for fragment analysis.

## Authors' contributions

FL and EB jointly conceived of the study. ACW carried out most of the practical molecular part of the study supervised by FL. ACW drafted the manuscript in collaboration with FL, EOR and EB. All authors read and approved the final manuscript.
